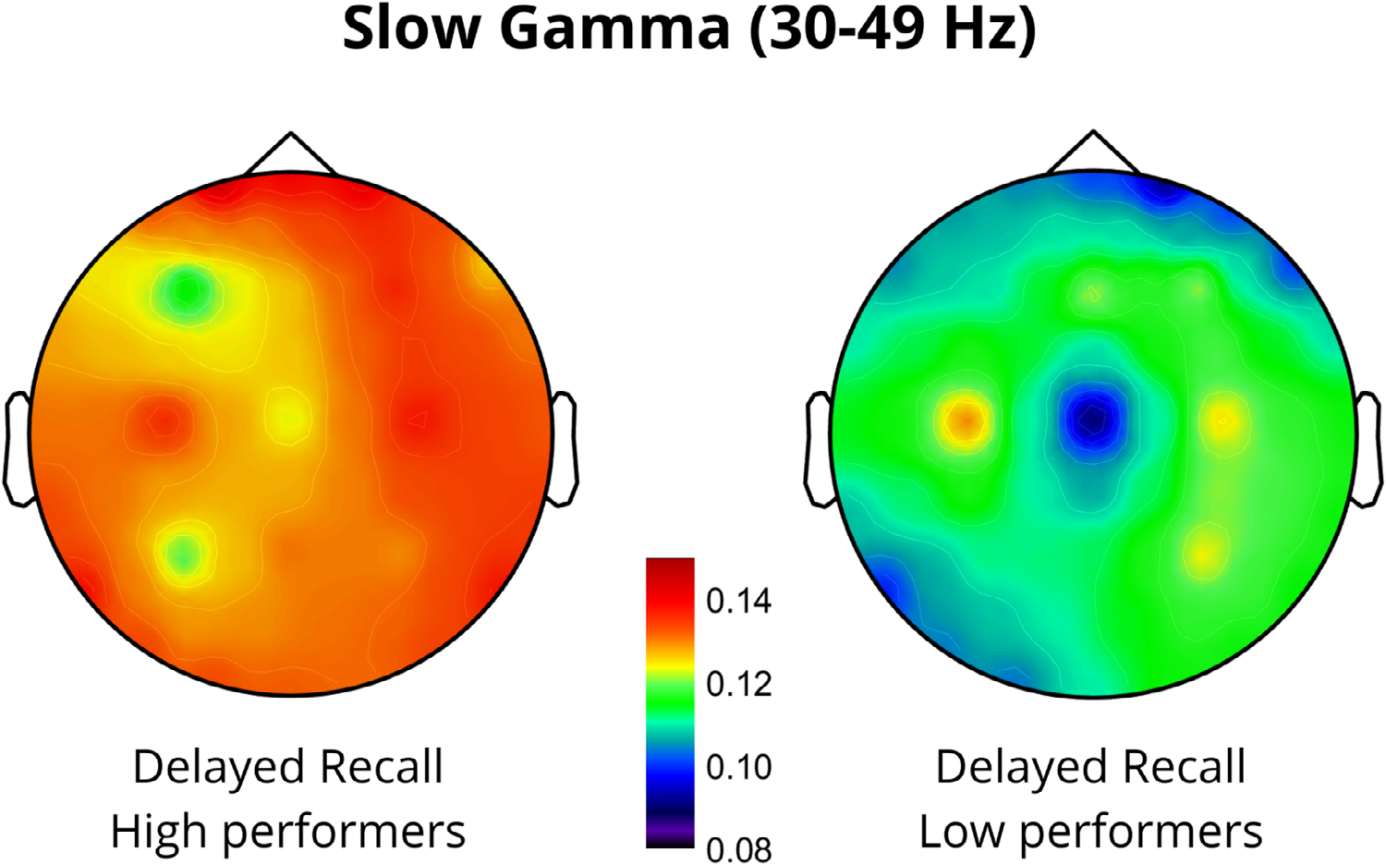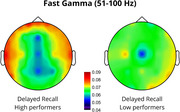# Linking resting‐state gamma activity to associative memory: a potential biomarker for cognitive aging and Alzheimer's Disease

**DOI:** 10.1002/alz70856_102676

**Published:** 2025-12-25

**Authors:** Giorgia Francesca Scaramuzzi, Valerio Manippa, Gaetano Scianatico, Ester Cornacchia, Aurora Bonvino, Daphne Gasparre, Davide Rivolta, Paolo Taurisano

**Affiliations:** ^1^ University of Bari Aldo Moro, Bari, Bari, Italy; ^2^ University of Bari “Aldo Moro”, Bari, Bari, Italy; ^3^ Department of Translational Biomedicine and Neuroscience “DiBraiN”, University of Bari Aldo Moro, Bari, Italy

## Abstract

**Background:**

Associative memory is a pivotal component of social cognition. The loss of this ability, frequently reported in the initial stages of Alzheimer's Disease (AD), is among the earliest indications of cognitive impairment. Recent studies have demonstrated that brain oscillations in the gamma band (γ, 30‐120 Hz) play a pivotal role in higher‐order cognitive functions, including multisensory integration (Senkowski et al., 2009) and memory consolidation (Fernandez‐Ruiz et al., 2021). This study aims to establish whether resting‐state EEG (rsEEG) spectral power can serve as a predictive biomarker of associative memory ability, possibly identifying early signs of memory challenges that may also be observed in AD patients.

**Method:**

Forty‐eight healthy adults underwent rsEEG recording, followed by a face‐name association task (FNAT; Manippa et al., 2025), assessing immediate (IR) and delayed recall (DR).

**Result:**

Results showed that endogenous slow‐gamma (s‐γ, 30–49 Hz) power significantly predicted DR, accounting for 22% of the variance (*p* = 0.024). Increased s‐γ power in temporal regions was positively associated with memory performance (*p* = 0.038). Fast‐gamma (f‐γ, 51–100 Hz) power significantly predicted DR, accounting for 27% of the variance (*p* = 0.006), with increased frontal (*p* = 0.045) and reduced posterior (*p* <0.001) f‐γ power predicting better performance. Lastly, a trend was observed where increased temporal f‐γ power and reduced posterior f‐γ power were associated with better IR performance.

**Conclusion:**

The correlation between increased temporal s‐γ and improved accuracy confirms temporal lobe involvement in the consolidation and retrieval of associative memories (Mayes et al., 2007), consistent with reports of reduced gamma power in the medial temporal lobe in AD patients (Babiloni et al., 2020). Moreover, increased frontal f‐γ activity suggests greater reliance on executive control processes, typically located in the prefrontal cortex, facilitating retrieval (Wang et al., 2018). Reduced posterior f‐γ activity may indicate a shift from sensory integration to higher‐order cognitive processing, with disruptions potentially reflecting imbalanced network dynamics, as observed in AD (Verret et al., 2012). These findings offer novel insights into the neurophysiological mechanisms underlying associative memory, possibly facilitating monitoring and prevention of cognitive decline in populations at risk for AD.